# The effect of reward and voluntary choice on the motor learning of serial reaction time task

**DOI:** 10.3389/fpsyg.2024.1493434

**Published:** 2025-01-07

**Authors:** Yanghui Quan, Jiayue Wang, Yandong Wang, Guanlan Kang

**Affiliations:** ^1^School of Psychology, Beijing Sport University, Beijing, China; ^2^Laboratory of Sports Stress and Adaptation of General Administration of Sport, Beijing Sport University, Beijing, China

**Keywords:** motor learning, reward, voluntary choice, motivation, serial reaction time task

## Abstract

**Objective:**

Reward and voluntary choice facilitate motor skill learning through motivation. However, it remains unclear how their combination influences motor skill learning. The purpose of the present study is to investigate the effects of reward and voluntary choice on motor skill learning in a serial reaction time task (SRTT).

**Methods:**

Participants completed six parts of SRTT, including pre-test, training phase, immediate post-test, a random session, delayed post-test, and retention test on the following day. During the training phase, participants were divided into four groups (reward_choice, reward_no-choice, no-reward_choice, no-reward_no-choice). In the reward condition, participants received reward for correct and faster (than a baseline) responses while those in the no-reward groups did not. For the choice manipulation, participants in the voluntary choice groups chose the color of the target, whereas in the forced choice groups, the same color was assigned by the computer.

**Results:**

The results showed that the four groups did not exhibit any significant differences in reaction time and error rate in the pre-test phase. Importantly, both reward and voluntary choice significantly enhanced sequence-specific learning effects, while no interaction was found. No significant effects of reward and voluntary choice were observed in the retention test.

**Conclusions:**

These findings suggest that reward and voluntary choice enhance motor skill performance and training independently, potentially at the action-selection level, which implies different mechanisms underlying the influences of reward and voluntary choice.

## 1 Introduction

Motor skill learning is crucial to human adaptation and development, as it is involved in a wide range of activities, including but not limited to typing, driving vehicles, and playing sports. Motor skill learning is characterized by a number of fundamental features, such as optimal movement selection and execution, improved movement speed and accuracy, as well as decreased movement variability and error (Yadav and Duque, 2023). Action selection and execution are two essential procedural components of motor skill learning (Diedrichsen and Kornysheva, 2015). Action selection involves the decision-making process where an individual chooses the most appropriate action from a set of possible actions (Kim et al., 2021). Execution, conversely, is the specification of actual muscle commands. Among the numerous paradigms of studying motor skill learning, the serial reaction time task (SRTT) specifically focuses on the action-selection level (Diedrichsen and Kornysheva, 2015; Rowland and Shanks, 2006). During SRTT, participants need to press the corresponding key according to the target location as quickly as possible. Reward and voluntary choice were often used to improve motor skill learning (for reviews, see Wulf and Lewthwaite, 2016; Zhao et al., 2024). However, little is known about the interaction or combining effect of reward and voluntary choice on motor skill performance and training.

External rewards, such as money and praise, are usually used in studies investigating the reward effect on a series of cognitive processes, such as attentional selection, conflict control and action et al. (Chelazzi et al., 2013; Kang et al., 2019, 2024; Grehl et al., 2022; Martinez et al., 2024; Sugawara et al., 2012; Vassiliadis et al., 2021). Previous studies have shown that participants who received rewards were more engaged in tasks and had better skill acquisition (Anderson et al., 2020; Palminteri et al., 2011; Vassiliadis et al., 2021; Wächter et al., 2009) and consolidation (Abe et al., 2011; Vassiliadis et al., 2021). For example, Wächter et al. (2009) investigated the differential impact of reward and punishment on motor learning. They trained participants on SRTT and found that the reward group showed more significant learning of sequence than the punished and control groups, as evidenced by greater reaction time (RT) savings when comparing the sequence block with random blocks. A recent study used the pinch-grip force task to examine the effect of reward on motor learning and revealed that compared to providing feedback alone, training with reward markedly enhanced skill performance and consolidation (Vassiliadis et al., 2021). These findings are consistent with reinforcement theory suggesting that rewarded behavior tends to be reinforced and repeated over time to increase the frequency of achieving rewarding outcomes in the future (Schultz, 2006). Nonetheless, Bacelar et al. (2020) investigated the influence of reward and punishment on action selection and action execution and did not find differences between the reward (or punishment) group and the neutral group. In the task of Bacelar et al. (2020), the reward or punishment depended on a skill-irrelevant choice (a visual category task), which may influence the effects of reward and punishment. In the task of Bacelar et al. (2020), participants performed a category-learning task (action selection) followed by a golf-putting task (action execution). The reward or punishment on action selection was dependent on a skill-irrelevant choice (i.e., choosing the correct target in the category-learning task), which may influence the effects of reward and punishment. It is worth noting that Bacelar et al. (2020) tried to examine the effects of reward and punishment on action selection and action execution that were different from the two levels of skill learning (action selection and action execution) proposed by Diedrichsen and Kornysheva (2015), in which action selection activated appropriate spatiotemporal pattern of muscle activity for following execution. The influence of reward effect on motor skill learning, especially action selection and action execution, needs further investigation.

Voluntary choice was often used to elicit autonomy, which is an important factor in motor skill performance and learning (Sanli et al., 2013). Previous studies have demonstrated that allowing participants to make their own choices regarding the practice variables, such as the use of assistive devices, practice schedule and the receipt of feedback could be helpful in improving their subsequent performance (Keetch and Lee, 2007; Post et al., 2011; Wulf et al., 2001). For example, when learning a basketball set shot, the self-control group could freely decide the amount of practice and the spacing between each shot, while the yoked group was matched with their counterparts in the self-control group in terms of practice schedule. The former showed a higher accuracy in the retention test (Post et al., 2014). Carter et al. (2014) revisited earlier findings on self-controlled feedback schedules, comparing different timing strategies for feedback requests in a task. They found that participants who made feedback decisions after a trial or both before and after a trial performed significantly better in retention and transfer tasks than those who made decisions before a trial or in the yoked groups. Furthermore, this facilitating effect was also observed when the choice was irrelevant to the task (Grand et al., 2017; Lewthwaite et al., 2015; Wulf et al., 2018). For example, Lewthwaite and colleagues conducted two experiments to investigate whether task-irrelevant choices could facilitate motor skill learning (Lewthwaite et al., 2015). In their study, one group could choose the color of golf balls before a golf-putting task (Experiment 1) or choose which painting to hang on the wall before a balance task (Experiment 2), while the control group was yoked to choices made by the choice group. The results showed that the choice group exhibited superior skill performance and retention in both tasks. One possible explanation is that making voluntary choices helps individuals gain control over the external environment, which satisfies their psychological need of autonomy (Deci and Ryan, 1987; Ryan and Deci, 2000). The OPTIMAL (Optimizing Performance through Intrinsic Motivation and Attention for Learning) theory, proposed by Wulf and Lewthwaite (2016), provides a framework to elucidate how autonomy influences motor skill learning. The theory suggests that autonomy can strengthen the goal-action coupling not only directly but through enhanced expectancies, which increase self-efficacy and intrinsic motivation, facilitating motor performance and learning.

Although there was evidence supporting the positive effect of voluntary choice on motor skill learning, the effect of voluntary choice was limited (McKay et al., 2023, 2022; Parma et al., 2024). McKay et al. (2022) included 73 articles on self-controlled learning and detected a small effect of self-controlled practice (*g* = −0.11 to 0.26) after controlling for selection bias, suggesting a negligible distinction between self-controlled and yoked practice conditions. Additionally, some empirical studies with large sample sizes have failed to observe the learning benefit of self-controlled practice (e.g., McKay and Ste-Marie, 2020a; St. Germain et al., 2023, 2022). For example, St. Germain et al. (2022) manipulated both choice availability and feedback characteristics in a handle-moving task. The results indicated that groups with the opportunity to choose when to receive feedback did not show any significant reduction in spatial and timing error compared to yoked groups. They concluded that contrary to voluntary choice, feedback was more critical for skill acquisition in a motor adaptation task, as it may have provided sufficient information for movement modulation relative to the task goal. However, it is important to note that choosing feedback schedule is different from choosing incidental things (such as the color of a ball). Feedback is directly related to performance-based adjustment and has a significant impact on an individual's expectancies. The present study mainly focused on the selection of task-irrelevant stimuli.

Furthermore, previous research investigating the impact of voluntary choice on motor skill learning has predominantly focused on the execution level, for example, golf-putting (An et al., 2020; McKay and Ste-Marie, 2020a), dart-throwing (Ikudome et al., 2019; McKay and Ste-Marie, 2020b), while its effects on the selection level remain underexplored. Recent studies have shown that voluntary choice can facilitate cognitive processing (Luo et al., 2022, 2024). Recent studies have shown that voluntary choice can facilitate performance on cognitive tasks. For instance, in a study by Luo et al. (2022), participants could either freely choose a picture (in the voluntary choice condition) or choose the selected picture (in the forced choice condition) as the task background before completing a visual search task. The results showed a reduced RT in subsequent task performance following a voluntary choice compared to a forced choice. The authors suggested that the belief of control from choice-outcome causation had a general facilitating effect on the process of response. Considering that action selection is essentially a cognitive process, whether and how voluntary choice influences the action-selection level of motor skill performance and training remain further studied.

The purpose of the present study was to examine the combined effect of reward and voluntary choice on motor skill learning in a serial reaction time task. The experiment was conducted on two consecutive days comprising six parts: pre-test, training, immediate post-test, a random session, delayed post-test, and retention test on day 2. The experimental procedure was adapted from previous studies (Doppler et al., 2019). During the training phase, we manipulated both reward and voluntary choice which combined to form four training conditions (i.e., reward_choice, reward_no-choice, no-reward_choice, no-reward_no-choice), and participants were assigned to these four groups accordingly. Specifically, participants in the reward groups received performance-dependent monetary rewards, while participants in the no-reward groups did not receive any rewards. Participants in the voluntary choice groups could choose the color of the target stimulus before starting each 12-trial training, while the color was predetermined for those in the no-choice groups, which matched their counterparts in the voluntary choice groups. Two important dependent variables index SRTT performance and learning were calculated, that is, general learning (GL) and sequence-specific learning (SSL) (Dovern et al., 2011; Meier and Cock, 2014). Specifically, GL effect indicates performance improvement due to repetitive practice in motor response, usually indexed by the RT difference between the pre-test and immediate post-test. SSL indicates performance improvement due to the acquisition of implicit, sequence-specific knowledge. In the current experimental procedure, since the random session and the immediate post-test achieve the same level of learning in motor response (i.e., general learning), the RT difference between the random session and the immediate post-test session may indicate SSL. However, the increased RT in the random session may be due to participants' fatigue, so a delayed post-test was added to control for the potential fatigue effects. Thus, SSL is calculated by the RT difference between the random session and the mean of two post-tests. In accordance with previous studies showing that reward expectation facilitates goal-directed task performance, we predicted that reward would improve SRTT performance in the training phase and post-tests as measured by GL and SSL. Previous studies on task-irrelevant choice demonstrated that while choice did not facilitate performance during practice, it did benefit skill retention or transfer (Iwatsuki and Otten, 2020; Lewthwaite et al., 2015; Wulf et al., 2014). Therefore, we expected that voluntary choice would facilitate skill retention. Moreover, the OPTIMAL illustrates that extrinsic reward and autonomy can reinforce the goal-action coupling, maintaining a focus on the task goal and reducing a self-focus, which leads to enhanced motor performance and learning (Wulf and Lewthwaite, 2016). We hypothesized that a combination of reward and voluntary choice would positively influence the performance and learning of the SRTT.

## 2 Methods

### 2.1 Participants

A power analysis was conducted using the option ANOVA: fixed effects, special, main effects and interactions in G^*^Power 3.1 with the following parameters: effect size *f* = 0.27, α = 0.05, β = 0.20, numerator *df* = 1, and number of groups = 4. The analysis revealed a total sample size of 110. The chosen effect size was according to a meta-analysis of published self-controlled learning experiments, which reported a moderate benefit of self-controlled practice, *g* = 0.54 (McKay et al., 2022). Although the effect size was slightly higher than the overall estimate (*g* = 0.44) of McKay et al. (2022), it was still used to calculate the sample size because the empirical studies (e.g., Lewthwaite et al., 2015; Post et al., 2014; Wulf et al., 2018) cited in the present study showed a moderate to high effect sizes for choice (*g* > 0.7) and research funding was limited. The present study recruited 119 participants (23 males, *M*_age_ = 20.05, SD = 2.32) in total from universities in Beijing. Participants were randomly assigned into four groups, that is, a reward_ choice group (RC), a reward_no-choice group (RNC), a no-reward_choice group (NRC), and a no-reward_no-choice group (NRNC). Five participants' error rate went beyond 3 SD of the average error rate, two participants did not attend the retention test, and two participants failed to complete the questionnaire. Therefore, they were all removed from the data analysis. Finally, 110 participants were included in the data analysis (RC group: *n* = 26, 5 males, *M*_age_ = 20.27, SD = 2.48; RNC group: *n* = 27, 6 males, *M*_age_ = 19.70, SD = 1.77; NRC group: *n* = 28, 6 males, *M*_age_ = 20.43, SD = 2.91; NRNC group: *n* = 29, 6 males, *M*_age_ = 20.05, SD = 2.02). All the participants were right-handed, had normal or corrected-to-normal vision, and were free from neurological/physical disorders. The present study was approved by the ethics committee of Beijing Sport University. All participants were financially compensated at the end of the experiment.

### 2.2 Materials and equipment

Two fixed sequences with 12 elements were used in the present study: 3–4–2–3–1–2–1–4–3–2–4–1 (A sequence) and 1-2-1-4-2-3-4-1-3-2-4-3 (B sequence), where the numbers 1–4 represented four stimulus locations from left to right. These sequences were used in the previous study and were proved equal (Bo et al., 2011). The visual stimuli were presented on a 14.2-inch computer screen (refresh rate: 60 Hz). Experiments were run with Psychopy 2.1.2 (Peirce, 2007).

### 2.3 Procedure

The formal experiment consisted of six stages across 10 sessions: s1-S2-S3-S4-S5-S6-s7-r8-s9-s10. Specifically, s1 denoted the pre-test, S2-S6 denoted training sessions, s7 denoted the immediate post-test, r8 denoted a random session, s9 denoted the delayed post-test, and s10 denoted the retention test after 24 h. Within each session, a 12-element sequence was repeated six times, resulting in 72 trials per session. Participants would learn either sequence A or sequence B, which was presented in fixed-sequence sessions (sessions with the letter “s” or “S”). In each experimental group, half of the participants were assigned to learn sequence A, and the other half were assigned to learn sequence B. To prevent the development of explicit knowledge of the sequences, each fixed session started at a unique position within the sequence. In the random session (r8), the sequence was randomly generated.

In the pre-test, post-test, and retention test phases, all the participants completed the traditional version of SRTT without any feedback. At the beginning of each trial, four blank squares with white borders were displayed in a line in the center of the screen for 250 ms. Then one of the squares turned white, and participants were required to respond as quickly and accurately as possible by pressing the corresponding key (D: left middle finger, F: left index finger, J: right index finger, K: right middle finger). The target disappeared after the key press or lasted for 1,000 ms. There was an interval of 250 ms between each trial.

In the training phase, the choice phases and feedback phases were added to the SRTT for the manipulation of reward and voluntary choice. Participants were randomly assigned into four groups and trained under different experimental conditions. The training phase consisted of five sessions, with a sequence repeated six times per session. The choice phase was presented before the beginning of each 12-trial training session, resulting in a total of 30 times of occurrence until the end of the training phase. Specifically, a fixation point appeared in the center of the screen for 400 ms. Participants in the voluntary choice groups were instructed that in the next part, they could freely select a color by pressing the corresponding key (“R” for the left, “U” for the right). In contrast, participants in the forced choice groups were informed that the computer would randomly select a color for them. After the instruction, two squares with complementary colors (e.g., blue and yellow) were horizontally displayed on the screen. For participants in the voluntary choice groups, the time allowed for making choices was unlimited, and the same amount of time was used for computer selection in the forced choice groups. The selected color (e.g., blue) was displayed in the center of the screen for 1,000 ms and used as the target color in the following 12 trials.

**Figure 1:**
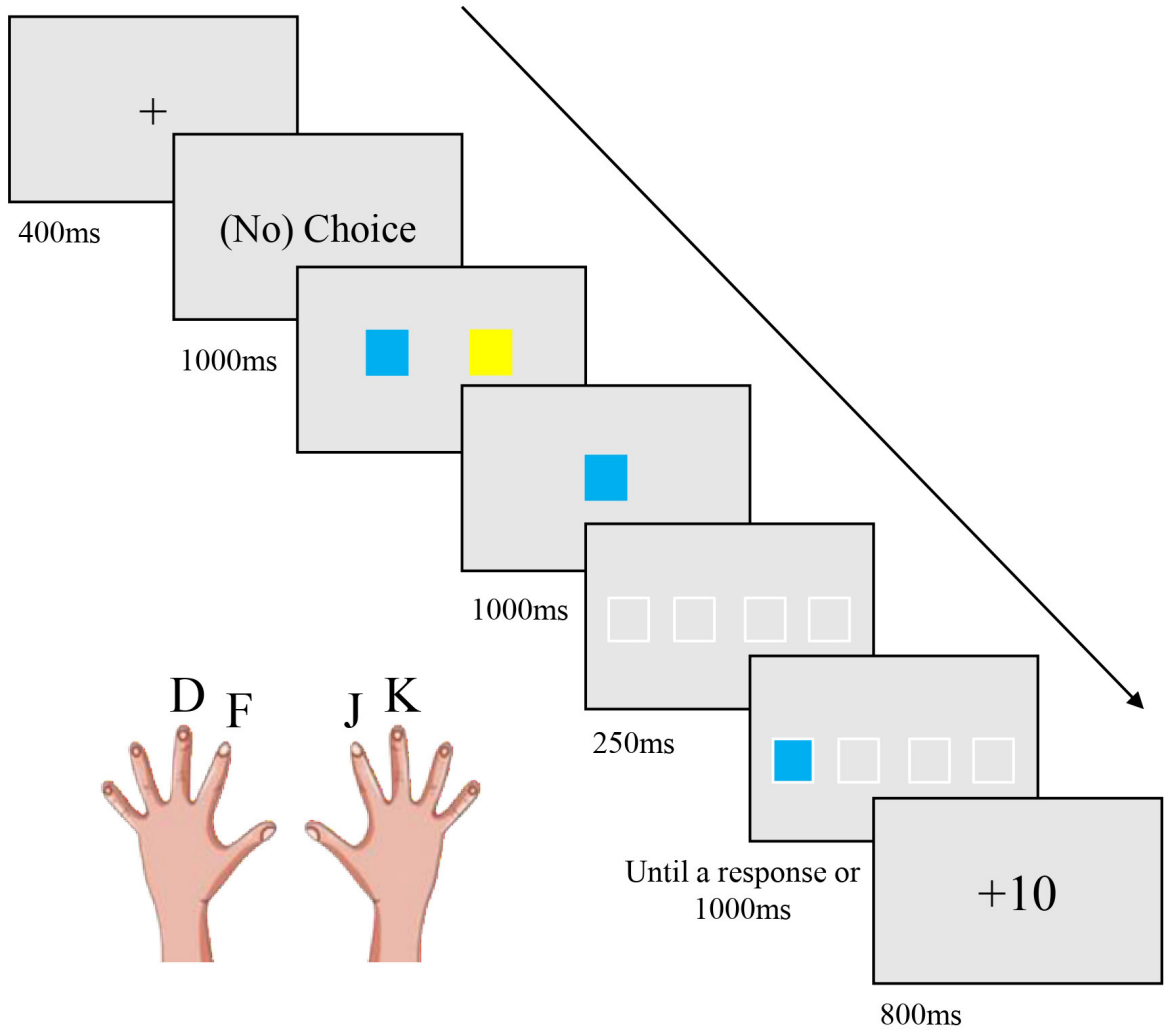
A rewarded trial with the choice phase during the training phase. The choice phases and feedback phases were added to the SRTT for the manipulation of reward and voluntary choice in the training phase.

For the manipulation of reward, participants in the reward groups were informed that the points gained in the training sessions would be converted into their final pay at the end of the experiment, while participants in the no-reward groups were informed that no reward would be given in the experiment. If the response was correct and faster than individual criterion RT, positive feedback was presented following the key press for 800 ms (“+10” for the reward groups, “+0” for the no-reward groups). The criterion RT was calculated as the mean RT in the pre-test. “Correct, too slow” was displayed when the response was correct but slower than the criterion RT. “Too slow!” was displayed when the key press was not made within 1,000 ms. “Wrong” was displayed if a key press error occurred. Considering the limited research funding and the potential negative impact of a small exchange rate on participants' motivation, participants in the reward groups were not informed of the specific exchange rate between points and money, and the total points they gained in the training sessions. After the experiment, the experimenter randomly selected one reward amount from 4, 5, or 6 (Chinese yuan), which was added to the basic payment (10 Chinese yuan). All participants accepted the final experimental payment without any questions. Figure 1 depicted a rewarded trial with the choice phase.

After the experiment, participants were required to complete a questionnaire consisting of five questions according to their feelings during the experiment. Participants rated on these items (1–7 indicated “entirely disagree – entirely agree”):

1. “I had the power to choose throughout the experiment”;
2. “I was fully capable of the task”;
3. “This task was very interesting”;
4. “I was very satisfied with my performance throughout the experiment”;
5. “I was nervous throughout the experiment.”

### 2.4 Statistical analysis

The error rate was calculated as the proportion of incorrect trials and omissions. The mean error rate was 2.5% (SD = 2.2). ANOVAs conducted on error rates showed no significant effect for each session (*p*s > 0.1). Therefore, we focused on the analysis of RTs. For the analysis of correct RTs, trials with RT beyond 3 SD of the mean of each participant were excluded. Finally, 96.1% of trials were included in the statistical analysis of RTs.

Firstly, 2 (Reward: reward vs. no-reward) × 2 (Choice: choice vs. no-choice) ANOVAs were conducted on the mean RTs of the pre-test to determine if participants in the four conditions exhibited differences. Secondly, to examine the effects of reward and voluntary choice in the training session, we conducted a 2 (Reward) × 2 (Choice) × 5 (training sessions) repeated-measures ANOVA on RT. To further evaluate the training effect, we performed linear regressions across the five training sessions and extracted the slope of the regression fits for each participant to establish the learning rates. The slope values were analyzed using a two-way ANOVA (Reward × Choice). Thirdly, to explore the impact of reward and choice on motor skill performance, we explored GL, SSL, and retention effects by two-way ANOVAs. The GL effect was calculated by the RT difference between s1 and s7. The SSL effect was determined as the RT difference between r8 and the mean of s7 and s9. The retention effect was calculated by comparing s10 to s1. Finally, for analyses of the subjective report, two-way ANOVAs were conducted on each item of the questionnaire. Statistical analyses were performed using IBM SPSS 23.0 (IBMCorp., Armonk, N.Y., USA), and the alpha level was set at 0.05.

## 3 Results

### 3.1 Behavioral data

A two-way ANOVA conducted on mean RT of the pre-test did not show any significant main effects or interaction, *ps* > 0.16, suggesting that initial performance was comparable in different groups.

**Figure 2:**
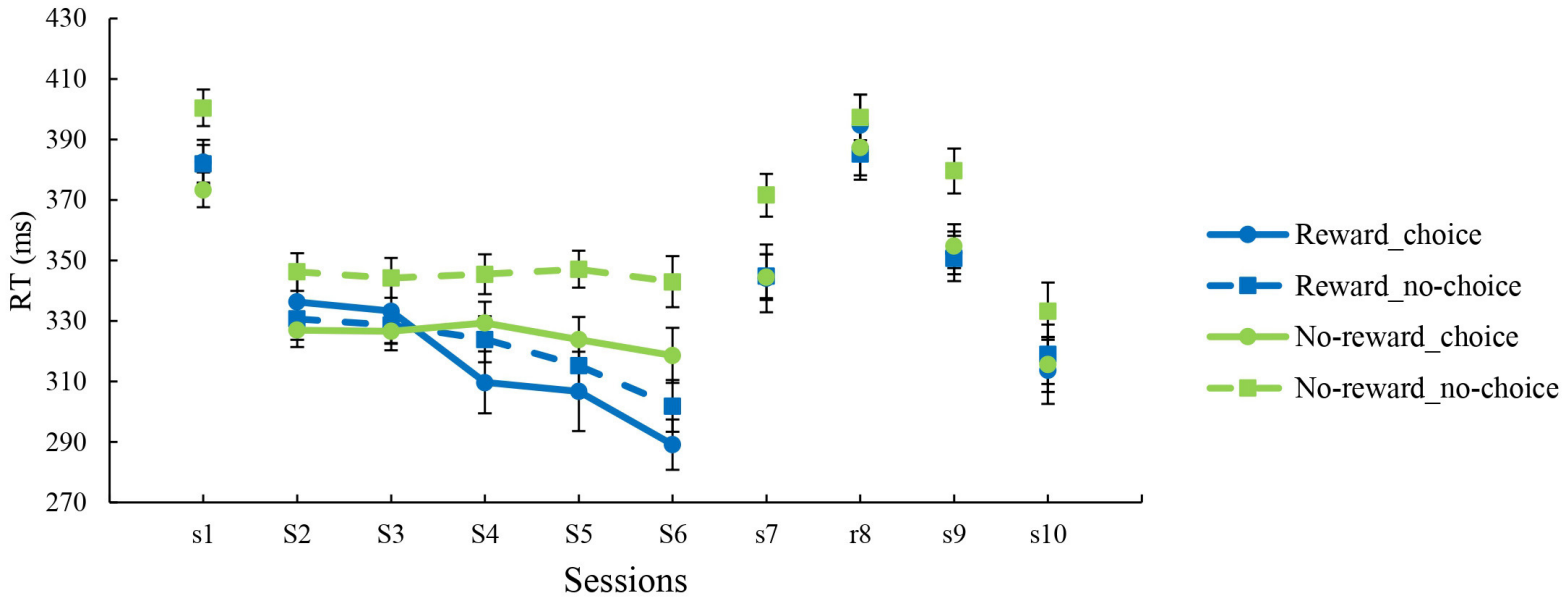
Mean reaction time for each group across all sessions. Displayed are the means ± SEM.

To examine the effects of reward and choice on the training phase, a repeated-measures ANOVA with reward and choice as between-subject factors and training session as a within-subject factor was conducted. The results revealed significant main effects of session, *F*(4, 103) = 6.773, *p* < 0.001, $\eta_{p}^{2}$ = 0.208, reward, *F*(1, 106) = 6.864, *p* = 0.010, $\eta_{p}^{2}$ = 0.061 and the interaction of reward and session, *F*(4, 103) = 4.041, *p* = 0.004, $\eta_{p}^{2}$ = 0.136. The main effect of choice was not significant, *F*(1, 106) = 3.524, *p* = 0.063, $\eta_{p}^{2}$ = 032. Simple effects analysis showed that only the reward groups showed significantly reduced RTs from S2 to S4, S5, and S6 (*ps* < 0.001), whereas no significant differences in the no-reward groups across the training process (*ps* > 0.1). In order to gain further insight into the training rates, linear regressions were performed on RTs obtained in five training blocks, and the slope of the fits was extracted for each participant. The slope values represented the rates of performance improvement and were analyzed using a 2 (Reward) × 2 (Choice) ANOVA. The results showed a significant main effect of reward, *F*(1, 106) = 15.276, *p* < 0.001, $\eta_{p}^{2}$ = 0.126. The slopes were steeper in the reward groups than in the no-reward groups, indicating that the reward groups improved faster than the no-reward groups. The main effect of choice and the interaction were not significant (*ps* > 0.13). Additionally, no differences were observed when comparing the intercepts of the reward and no-reward groups (*p* = 0.078), which indicated that the reward-induced training effect could not be explained by initial performance. Figure 2 illustrated the mean RT of each session for all groups.

**Figure 3:**
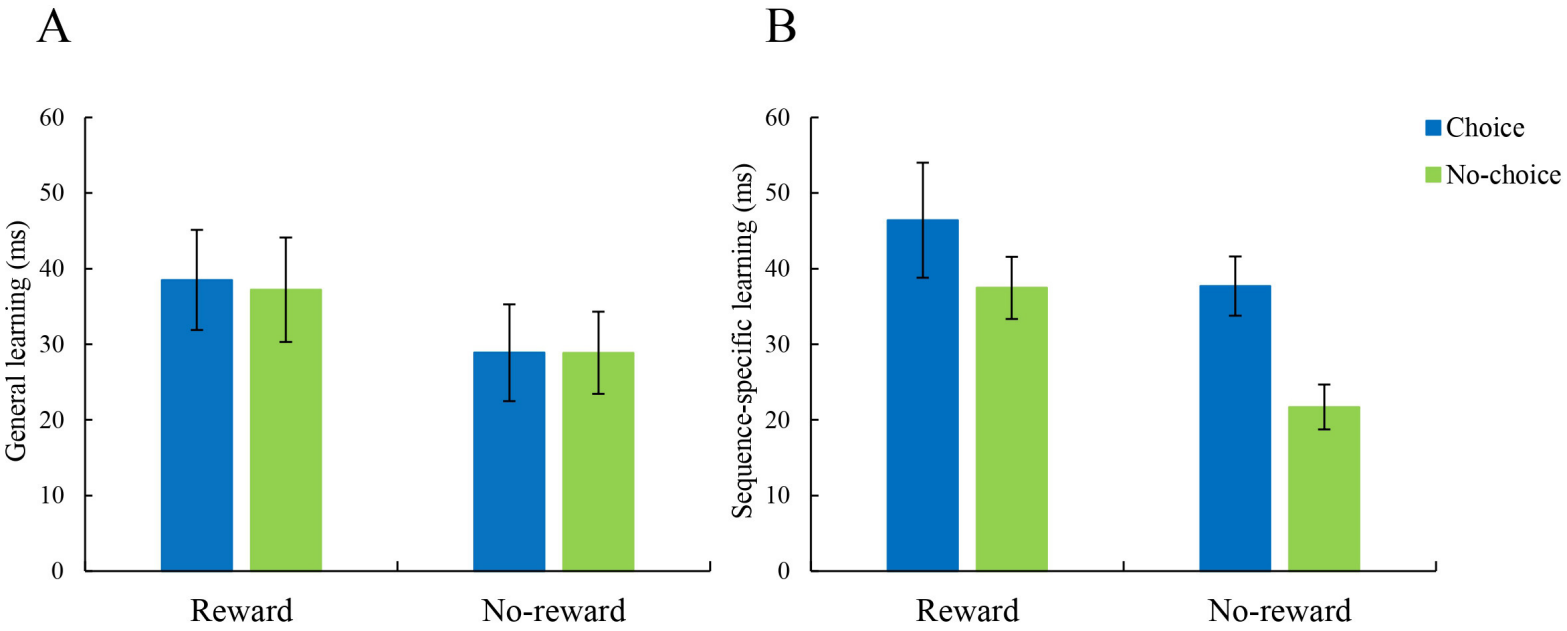
**(A)** General learning effects under different conditions. No main effects or interaction was observed. The choice condition is shown in blue, the no-choice condition is shown in green. **(B)** Sequence-specific learning effects under different conditions. There was a significant main effect of reward and voluntary choice on SSL, indicating that the reward groups gained more sequence-specific learning effects than the no-reward groups and that the choice groups gained more sequence-specific learning effects than the no-choice groups. No interaction of reward and choice was observed. Displayed are the means ± SEM.

For the general learning effect (see Figure 3A), no main effects and interaction were observed (*ps* > 0.15). For the sequence-specific learning effect (see Figure 3B), a main effect of reward was found, *F*(1, 106) = 6.404, *p* = 0.013, $\eta_{p}^{2}$ = 0.057, indicating that the reward groups have more sequence-specific enhancement than the no-reward groups. The main effect of choice was significant, *F*(1, 106) = 6.590, *p* = 0.012, $\eta_{p}^{2}$ = 0.059, indicating that the choice groups have more sequence-specific enhancement than the no-choice groups. The interaction did not reach significance (*p* = 0.456). These results indicate that both reward and choice enhanced sequence-specific learning, but these two factors may work independently. Regarding the retention effect on day 2, no significant main effects or interaction were observed, *ps* > 0.28.

### 3.2 Subjective report

Participants in the choice groups reported higher autonomy than participants in the no-choice groups, evidenced by a main effect of choice on item 1, *F*(1, 106) = 39.306, *p* < 0.001, $\eta_{p}^{2}$ = 0.271, indicating the effective manipulation of choice. No significant effects of reward, choice, or their interaction were found on item 2 (*ps* > 0.31) or item 3 (*ps* > 0.14). A significant Reward × Choice interaction was found on item 4, *F*(1, 106) = 4.240, *p* = 0.042, $\eta_{p}^{2}$ = 0.038. Simple effects analysis showed that participants in the choice group were marginally more satisfied with their performance than participants in the no-choice group under the reward condition (5.54 ± 0.25 vs. 4.89 ± 0.24, *p* = 0.064), while no significant difference in the no-reward condition (*p* = 0.223). A significant Choice × Reward interaction on item 5 was observed, *F*(1,106) = 5.605, *p* = 0.020, $\eta_{\text{p}}^{2}$ = 0.050. Specifically, under the reward condition, participants with choice felt significantly less nervous than participants with no choice (2.85 ± 0.30 vs. 3.81 ± 0.30, *p* = 0.025), while there was no significant difference on nervousness under the no-reward condition (*p* = 0.296).

## 4 Discussion

In the present study, we employed the SRTT to investigate the impact of reward and voluntary choice on motor skill performance and learning. During the training phase, participants received manipulations of reward and voluntary choice. The results indicated that reward and voluntary choice significantly enhanced the sequence-specific learning effect, yet no interaction was observed. Neither reward nor voluntary choice affected GL and retention with an interval of 24 h. These findings suggest that reward and voluntary choice may benefit motor skill performance at the action-selection level through independent ways, which implies different mechanisms underlying the influences of reward and voluntary choice.

The facilitating effect of reward was found on the sequence-specific learning effect, which was consistent with the findings of Wächter et al. (2009). In the present study, the reward groups received performance-contingent monetary rewards during the training phase. The beneficial effect of reward was observed in the training phase, and extended to post-training performance indexed by sequence-specific learning. Although the performance of the SRTT is often constrained by speed-accuracy trade-off, reward has been demonstrated to be a strong motivational factor, which could improve both speed and accuracy of movements (i.e., better performance) (Manohar et al., 2015). Evidence from neuroimaging studies showed that individuals exhibited stronger neural activity in striatum during motor skill training with reward (Doppler et al., 2019; Widmer et al., 2016). Such reward modulation on motor adjustments has been shown to be dependent on dopamine (Schultz, 1998), a key neurotransmitter that carries the reward signal. Experiencing reward as well as the desire for rewarding stimuli, elicits dopamine activity, which directs attention to valuable cues and induces motivation to obtain reward (Ferguson et al., 2020; Knowlton and Castel, 2022; Schultz, 2010, 2013). However, reward did not enhance the general learning effect. General learning indicated faster response irrespective of sequence structure. In contrast, sequence-specific learning encompassed both implicit sequence knowledge and key-pressing execution. In the post-tests participants performed without reward. Therefore, it is possible that reaction-based performance (i.e., general learning) decayed since each fast response was not reinforced in time, while sequence-based performance (i.e., sequence-specific learning) was improved since rewarded participants may have encoded sequence knowledge during the training phase.

Although reward boosted immediate skill performance, it did not enhance retention, which aligns with previous studies on the SRTT (Doppler et al., 2019; Steel et al., 2016). Retention of motor skills is influenced by multiple factors, including the length of the retention interval, activities performed during the interval and sleep (Cohen et al., 2005). In the present study, we did not restrict the retention interval to exactly 24 h, nor did we control for the periods of sleep and wakefulness, or measure the quality of sleep and other activities during the interval, which may potentially affect the results of retention.

Voluntary choice was found to benefit the sequence-specific learning effect but had no impact on skill retention. Previous research has shown that following the making of a task-irrelevant choice, participants exhibited improved motor skill learning at the execution level indexed by enhanced performance in the retention and transfer test (Lewthwaite et al., 2015; Wulf et al., 2014). For example, in a ball-throwing task, participants who were given the opportunity to choose the ball color during practice showed higher throwing accuracy in retention and transfer tests than the control group (Wulf et al., 2014). Providing choice has been suggested to allow a feeling of autonomy, which enhances intrinsic motivation to focus on the task goal, leading to superior motor performance and learning (Wulf and Lewthwaite, 2016). However, our results showed that voluntary choice improved immediate motor performance on day 1, but had no facilitating effect on the skill retention. Skill retention was calculated by the difference between the first session on day 1 and the session on day 2, which were both sequence sessions. Thus, retention may only reflect response-based RT savings similar to general learning. Since the choice effect was not found on general learning, it may not extend to retention. Another possible explanation was that when the subsequent task primarily involves action execution that relies on muscle commands (e.g., golf-putting task), the effect of voluntary choice may be more pronounced and profound. In the present study, we used the SRTT, which mainly focuses on the selection process of motor skill learning (Diedrichsen and Kornysheva, 2015; Rowland and Shanks, 2006). Compared to action execution, action selection is more of a cognitive process involving complex internal mechanisms and higher-order information processing, which may be more susceptible to choice effect during online practice than offline consolidation.

Although both reward and voluntary choice facilitated sequence-specific learning, no combined effect of reward and voluntary choice was observed. It is possible that these two factors may influence the training processes in different ways. According to the results of the training phase, reward enhanced SRTT performance during the training phase. Receiving performance-dependent reward may reinforce accurate motor responses, thereby fostering motor-based implicit memory. Therefore, an effect of reward was observed on sequence-specific learning. Voluntary choice did not affect the SRTT performance during the training phase, whereas did have an impact on subsequent sequence-specific learning. In our study, participants in the choice groups could choose the color of target stimuli, which was irrelevant to the task, while participants in the no-choice groups were yoked to their counterparts in the choice groups. Autonomy provided by voluntary choice may increase task engagement and attention to the sequential stimuli, which may further positively influence the encoding of the sequence. Thus, an effect of voluntary choice was observed on sequence-specific learning.

Finally, a limitation of the current study is that motivation was not measured directly in any way before and after the experiment. It has been shown that the initial level of motivation affects the impact of voluntary choice on motor skill learning (Ikudome et al., 2019). In Ikudome and colleagues' study, participants were divided into two groups according to their levels of intrinsic motivation for the dart-throwing task in a preliminary experiment. In the formal experiment, some participants in each group could choose the dart color, while others were yoked to their counterparts. Results showed that voluntary choice had a positive effect on skill learning in the less motivated participants, but not in the highly motivated ones. Additionally, the lack of measurement on extrinsic and intrinsic motivation prevented the elucidation of the relative change of two types of motivation during the experiment. Further studies are needed to match the experimental groups based on motivation and to measure motivation levels to clarify the underlying mechanism. Another issue that needs to be addressed is that when calculating the sample size, we referred to an effect size estimated from only published self-controlled learning experiments (*g* = 0.54), which was slightly higher than the overall estimate (*g* = 0.44) reported by McKay et al. (2022). In the present study, the findings indicated moderate effect size of reward and choice ($\eta_{p}^{2}$ = 0.057–0.061), which was higher than the overall estimate, although lower than we expected. We included 110 participants in the final data analysis, with at least 26 participants in each experimental group. This was a reasonable sample size given that typically small sample sizes in motor learning studies (Lohse et al., 2016). Nevertheless, future studies should use more appropriate sample sizes to obtain reliable results.

In conclusion, the present study aimed to examine whether reward and voluntary choice have a combined effect on motor skill learning. The results demonstrated that reward and voluntary had positive impacts on sequence-specific learning, whereas no interaction was observed. Both reward and voluntary choice failed to benefit skill retention. These findings suggest that reward and voluntary choice enhance motor skill performance independently, potentially at the action-selection level, which implies different mechanisms underlying the influences of reward and voluntary choice.

## Data Availability

The datasets presented in this study can be found in online repositories. The names of the repository/repositories and accession number(s) can be found at: https://osf.io/ejhfr/.
